# Disordered LiZnVO_4_ with a phenacite structure

**DOI:** 10.1107/S1600536810013358

**Published:** 2010-04-17

**Authors:** Mohamed Azrour, Brahim Elouadi, Lahcen El Ammari

**Affiliations:** a Equipe Sciences des Matériaux, Faculté des Sciences et Techniques Errachidia, Morocco; b Laboratoire d’Elaboration, Analyse Chimique et Ingénierie, Département de Chimie, Université de La Rochelle, Avenue Marillac, 17042 La Rochelle Cedex 01, France; c Laboratoire de Chimie du Solide Appliquée, Faculté des Sciences, Université Mohammed V-Agdal, Avenue Ibn Battouta, BP 1014, Rabat, Morocco

## Abstract

Single crystals of lithium zinc vanadate, LiZnVO_4_, were grown by the flux method. The structural type of this vanadate is characterized by a three-dimensional arrangement of tetra­hedra sharing apices in an LiZnVO_4_ network. This arrangement contains three different tetra­hedra, namely one [VO_4_] and two disordered mixed-site [Li/ZnO_4_] tetra­hedra. The resulting lattice gives rise to hexa­gonal channels running along the [0001] direction. Both sites in the mixed-site [Li/ZnO_4_] tetra­hedra are occupied by a statistical mixture of lithium and zinc with a 1:1 ratio. Therefore, LiZnVO_4_ appears to be the first vanadate known to crystallize with a disordered phenacite structure. Moreover, the resulting values of calculated bond valences (Li = 1.083, Zn = 2.062 and V = 5.185) tend to confirm the structural model.

## Related literature

For related structural studies, see: Hartmann (1989 ▶); Capsoni *et al.* (2006 ▶); Zachariasen (1971 ▶). For compounds with the same structural type, see: Bu *et al.* (1996 ▶); Elammari & Elouadi (1989 ▶); Elouadi & Elammari (1990 ▶); Jensen *et al.* (1998 ▶). For bond-valence calculations, see: Brown & Altermatt (1985 ▶).

## Experimental

### 

#### Crystal data

LiZnVO_4_
*M* *_r_* = 187.25
Trigonal, 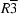

*a* = 14.107 (3) Å
*c* = 9.441 (2) Å
*V* = 1627.1 (6) Å^3^
*Z* = 18
Mo *K*α radiation
μ = 9.06 mm^−1^
*T* = 298 K
0.14 × 0.12 × 0.10 mm

#### Data collection

Bruker X8 APEXII CCD area-detector diffractometer
Absorption correction: multi-scan (*SADABS*; Sheldrick, 2003 ▶) *T*_min_ = 0.292, *T*_max_ = 0.404
9709 measured reflections
1622 independent reflections
1213 reflections with *I* > 2σ(*I*)
*R*_int_ = 0.070

#### Refinement

*R*[*F* ^2^ > 2σ(*F* ^2^)] = 0.024
*wR*(*F* ^2^) = 0.055
*S* = 1.04
1622 reflections
69 parameters
Δρ_max_ = 0.51 e Å^−3^
Δρ_min_ = −0.53 e Å^−3^

### 

Data collection: *APEX2* (Bruker, 2005 ▶); cell refinement: *SAINT* (Bruker, 2005 ▶); data reduction: *SAINT*; program(s) used to solve structure: *SHELXS97* (Sheldrick, 2008 ▶); program(s) used to refine structure: *SHELXL97* (Sheldrick, 2008 ▶); molecular graphics: *ORTEP-3 for Windows* (Farrugia, 1997 ▶) and *PLATON* (Spek, 2009 ▶); software used to prepare material for publication: *WinGX* (Farrugia, 1999 ▶).

## Supplementary Material

Crystal structure: contains datablocks global, I. DOI: 10.1107/S1600536810013358/fj2291sup1.cif

Structure factors: contains datablocks I. DOI: 10.1107/S1600536810013358/fj2291Isup2.hkl

Additional supplementary materials:  crystallographic information; 3D view; checkCIF report

## Figures and Tables

**Fig. 1.**
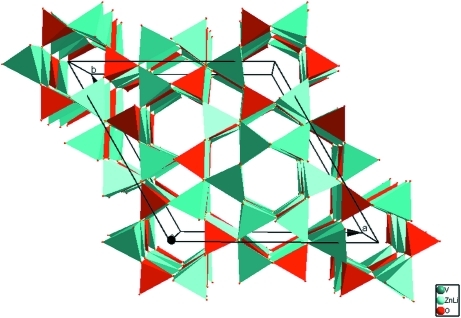
: A three-dimensional view of LiZnVO4 crystal structure, showing tunnels runnig along the c axis.

**Fig. 2.**
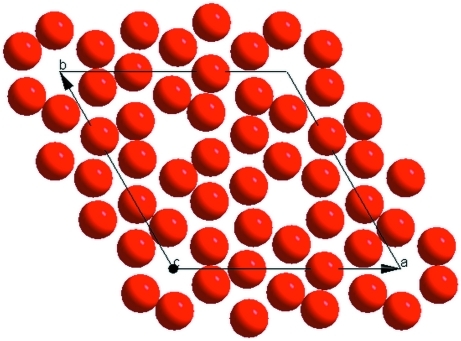
: Partial projection of the crystal structure on (0 0 1), showing lacunar hexagonal close packing of O2- ions.

## supplementary crystallographic information

### Comment

Our particular interest here is to investigate the form nature of the crystallized phase and determine the structural type that could result from the association of small size cations likely to enter under normal conditions of pressure and temperature, tetrahedral cavities of oxides like in phenacite Be_2_SiO_4_ network (Hartmann, 1989; Zachariasen, 1971). The compounds currently known to crystallize with such structural type are LiZnPO_4_ (Bu *et al.*, 1996; Elammari & Elouadi, 1989; Elouadi & Elammari, 1990) and LiZnAsO_4_ (Jensen *et al.*, 1998).

The structural type of the title compound, related to the phenacite structure, could be described (Fig.1) as three dimensional arrangement of [MO_4_] tetrahedra ( M= Li/Zn or V) sharing apices. The arrangement concerns three different types of tetrahedra [VO_4_] and two disordered sites [Li/ZnO_4_] which give rise to an overall disordered phenacite structure. When viewed along the c axis, the packing of [MO_4_] tetraherdra results in two types of tunnels: large hexagonal tunnels surrounded by six lozenge like channels (rings of four tetrahedra). Similar description has recently been reported by Capsoni *et al.* (2006) using a powder x-ray diffraction data of LiZnVO_4_. However, a careful observation of the two models can highlights the difference between our two results. Indeed, in addition to the difference of the lengths of chemical bonds, the occupancy rate of cationic Wycoff sites is different. Thus, in our model, there is only a disorder between Li and Zn with a statistical distribution of both ions on the two crystallographic sites, while the third site is only occupied by vanadium cation. Furthermore, A bond-valence analysis (Li <1.083>, Zn<2.062> and <V<5.185>) based on the empirical formula proposed by Brown & Altermatt (1985) is in favor of this model . The cationic disorder mentioned by Capsoni *et al.* could be seen as due to preparation methods. The powder used was slowly cooled from 853 K after 24 h sintering. Whereas, the growth of our crystal, from a flux melted at 1073 k and slowly cooled with a rate of 5 K h-1. Thus the resulting sintering of our crystal was much longer. A more ordred system is then to be expected.

When such structural type is seen as a close packing of oxygen anions, it appears as a lacunar hexagonal close packing of O^2-^ ions. Fig.2 shows a typical oxygen layer and the elevation of such oxygen plans as successively stacked ( ABAB···) along [0001]. The coordination sphere of all cations is of tetrahedral type. The analysis of oxygen environment shows a regular triangular cavity for O^2-^ anions with an average edge length of <V—Li/Zn> = 3.240 Å.

In the case of the present form of LiZnVO_4_, the disordered phenacite structure was attributed to the existence of a mixed tetrahedral site [Li/ZnO4] occupied by both Li and Zn. The resulting space group is R-3. LiZnVO_4_ is probably the first vanadate known to crystallize with a disordered phenacite structure.

### Experimental

Prior to the crystal growth, pulverulent samples of the compound LiZnVO_4_ and the flux LiVO_3_ are synthesized by the regular solid state reaction according to the following reactions:

Li_2_CO_3_ + 2ZnO + V_2_O_5_ —> 2LiZnVO_4_ + CO_2_ Li_2_CO_3_ + V_2_O_5_ —> 2LiVO_3_ + CO_2_

Single crystal of the monovanadate LiZnVO_4_ were grown from a bath of equimolar mixture of freohly prepared powders of LiZnVO_4_ and LiVO_3_. The starting mixture was thoroughly ground before to be melted at 1073 K in a platinum crucible and slowly cooled with a rate of 5 K h-1 to 773 K. The furnace was then switched off and the whole system naturally cooled down to room temperature. Single crystal s were collected from the crucible after dissolwing the flux in warmed water.

### Crystal data

**Table:**

LiZnVO_4_	*D*_x_ = 3.440 Mg m^−^^3^
*M**_r_* = 187.25	Mo *K*α radiation, λ = 0.71073 Å
Trigonal, *R*3	Cell parameters from 9709 reflections
Hall symbol: -R 3	θ = 10–30°
*a* = 14.107 (3) Å	µ = 9.06 mm^−^^1^
*c* = 9.441 (2) Å	*T* = 298 K
*V* = 1627.1 (6) Å^3^	Prism, pale yellow
*Z* = 18	0.14 × 0.12 × 0.10 mm
*F*(000) = 1584	

### Data collection

**Table:**

Bruker X8 APEXII CCD area-detector diffractometer	1622 independent reflections
Radiation source: fine-focus sealed tube	1213 reflections with *I* > 2σ(*I*)
graphite	*R*_int_ = 0.070
φ and ω scans	θ_max_ = 35.2°, θ_min_ = 4.0°
Absorption correction: multi-scan (*SADABS*; Sheldrick, 2003)	*h* = −22→22
*T*_min_ = 0.292, *T*_max_ = 0.404	*k* = −22→22
9709 measured reflections	*l* = −15→15

### Refinement

**Table:**

Refinement on *F*^2^	Primary atom site location: structure-invariant direct methods
Least-squares matrix: full	Secondary atom site location: difference Fourier map
*R*[*F*^2^ > 2σ(*F*^2^)] = 0.024	*w* = 1/[σ^2^(*F*_o_^2^) + (0.0124*P*)^2^ + 2.5069*P*] where *P* = (*F*_o_^2^ + 2*F*_c_^2^)/3
*wR*(*F*^2^) = 0.055	(Δ/σ)_max_ = 0.001
*S* = 1.04	Δρ_max_ = 0.51 e Å^−^^3^
1622 reflections	Δρ_min_ = −0.53 e Å^−^^3^
69 parameters	Extinction correction: *SHELXL97* (Sheldrick, 2008), Fc^*^=kFc[1+0.001xFc^2^λ^3^/sin(2θ)]^-1/4^
0 restraints	Extinction coefficient: 0.0088 (2)

### Special details

**Table:**

Geometry. All esds (except the esd in the dihedral angle between two l.s. planes) are estimated using the full covariance matrix. The cell esds are taken into account individually in the estimation of esds in distances, angles and torsion angles; correlations between esds in cell parameters are only used when they are defined by crystal symmetry. An approximate (isotropic) treatment of cell esds is used for estimating esds involving l.s. planes.
Refinement. Refinement on *F*^2^ for ALL reflections except for 0 with very negative *F*^2^ or flagged by the user for potential systematic errors. Weighted *R*-factors wR and all goodnesses of fit *S* are based on *F*^2^, conventional *R*-factors *R* are based on *F*, with *F* set to zero for negative *F*^2^. The observed criterion of *F*^2^ > σ(*F*^2^) is used only for calculating -*R*-factor-obs etc. and is not relevant to the choice of reflections for refinement. *R*-factors based on *F*^2^ are statistically about twice as large as those based on *F*, and *R*- factors based on ALL data will be even larger.

### Fractional atomic coordinates and isotropic or equivalent isotropic displacement parameters (Å^2^)

**Table:**

	*x*	*y*	*z*	*U*_iso_*/*U*_eq_	Occ. (<1)
V1	0.454581 (17)	0.138171 (16)	0.08352 (2)	0.00790 (7)	
Zn1	0.45273 (2)	0.14015 (2)	−0.24915 (3)	0.01132 (7)	0.50
Li1	0.45273 (2)	0.14015 (2)	−0.24915 (3)	0.01132 (7)	0.50
Zn2	0.64622 (2)	0.12175 (3)	0.24882 (3)	0.01150 (7)	0.50
Li2	0.64622 (2)	0.12175 (3)	0.24882 (3)	0.01150 (7)	0.50
O1	0.34102 (7)	0.01142 (7)	0.08427 (11)	0.01335 (17)	
O2	0.56475 (8)	0.11882 (9)	0.08215 (10)	0.01353 (17)	
O3	0.45578 (8)	0.20780 (8)	−0.06565 (10)	0.01419 (18)	
O4	0.45936 (8)	0.20728 (8)	0.23409 (10)	0.01377 (17)	

### Atomic displacement parameters (Å^2^)

**Table:**

	*U*^11^	*U*^22^	*U*^33^	*U*^12^	*U*^13^	*U*^23^
V1	0.00918 (10)	0.00812 (10)	0.00692 (9)	0.00472 (8)	−0.00012 (6)	−0.00004 (6)
Zn1	0.01190 (12)	0.01217 (13)	0.01038 (12)	0.00637 (10)	0.00052 (9)	0.00069 (9)
Li1	0.01190 (12)	0.01217 (13)	0.01038 (12)	0.00637 (10)	0.00052 (9)	0.00069 (9)
Zn2	0.01159 (13)	0.01368 (13)	0.01016 (12)	0.00701 (10)	0.00005 (9)	0.00037 (9)
Li2	0.01159 (13)	0.01368 (13)	0.01016 (12)	0.00701 (10)	0.00005 (9)	0.00037 (9)
O1	0.0109 (4)	0.0102 (4)	0.0180 (4)	0.0046 (3)	−0.0010 (3)	0.0000 (3)
O2	0.0121 (4)	0.0198 (4)	0.0115 (3)	0.0101 (3)	−0.0005 (3)	−0.0005 (3)
O3	0.0219 (5)	0.0120 (4)	0.0103 (3)	0.0096 (3)	−0.0007 (3)	0.0009 (3)
O4	0.0209 (4)	0.0125 (4)	0.0104 (4)	0.0102 (4)	0.0003 (3)	−0.0004 (3)

### Geometric parameters (Å, °)

**Table:**

V1—O1	1.7027 (10)	Zn2—O2	1.9368 (10)
V1—O4	1.7059 (10)	Zn2—O4^x^	1.9495 (10)
V1—O2	1.7071 (10)	Zn2—O4^xi^	1.9676 (11)
V1—O3	1.7123 (10)	Zn2—Li1^iv^	3.1441 (8)
V1—Li2^i^	3.1568 (7)	Zn2—Zn1^iv^	3.1441 (8)
V1—Li1^ii^	3.1719 (8)	Zn2—Li1^viii^	3.2314 (8)
V1—Li2^iii^	3.2409 (8)	Zn2—Li2^i^	3.2675 (7)
V1—Li1^iv^	3.2523 (6)	Zn2—Li2^x^	3.2676 (7)
V1—Li2^v^	3.2978 (7)	O1—Li2^iii^	1.9294 (11)
V1—Li1^vi^	3.3232 (7)	O1—Zn2^iii^	1.9294 (11)
Zn1—O2^vi^	1.9410 (10)	O1—Zn1^ii^	1.9442 (11)
Zn1—O1^vii^	1.9441 (11)	O1—Li1^ii^	1.9442 (11)
Zn1—O3^iv^	1.9588 (11)	O2—Li1^iv^	1.9411 (10)
Zn1—O3	1.9679 (11)	O2—Zn1^iv^	1.9411 (10)
Zn1—Zn2^vi^	3.1441 (8)	O3—Li1^vi^	1.9587 (11)
Zn1—Li2^vi^	3.1441 (8)	O3—Zn1^vi^	1.9587 (11)
Zn1—Li2^viii^	3.2314 (8)	O4—Li2^i^	1.9496 (10)
Zn1—Li1^vi^	3.2765 (7)	O4—Zn2^i^	1.9496 (10)
Zn1—Li1^iv^	3.2766 (7)	O4—Li2^v^	1.9676 (11)
Zn2—O1^ix^	1.9294 (11)	O4—Zn2^v^	1.9676 (11)
			
O1—V1—O4	110.14 (5)	O2^vi^—Zn1—O3	115.71 (4)
O1—V1—O2	106.61 (5)	O1^vii^—Zn1—O3	106.57 (4)
O4—V1—O2	108.58 (5)	O3^iv^—Zn1—O3	110.08 (5)
O1—V1—O3	109.88 (5)	O1^ix^—Zn2—O2	111.98 (5)
O4—V1—O3	111.80 (5)	O1^ix^—Zn2—O4^x^	108.76 (4)
O2—V1—O3	109.69 (5)	O2—Zn2—O4^x^	117.26 (4)
O2^vi^—Zn1—O1^vii^	109.39 (5)	O1^ix^—Zn2—O4^xi^	102.55 (4)
O2^vi^—Zn1—O3^iv^	106.12 (4)	O2—Zn2—O4^xi^	107.30 (4)
O1^vii^—Zn1—O3^iv^	108.84 (4)	O4^x^—Zn2—O4^xi^	107.87 (5)

Symmetry codes: (i) *y*+1/3, −*x*+*y*+2/3, −*z*+2/3; (ii) −*x*+*y*+2/3, −*x*+1/3, *z*+1/3; (iii) *x*−*y*−1/3, *x*−2/3, −*z*+1/3; (iv) *x*−*y*+1/3, *x*−1/3, −*z*−1/3; (v) −*x*+*y*+1, −*x*+1, *z*; (vi) *y*+1/3, −*x*+*y*+2/3, −*z*−1/3; (vii) −*y*+1/3, *x*−*y*−1/3, *z*−1/3; (viii) −*x*+1, −*y*, −*z*; (ix) *y*+2/3, −*x*+*y*+1/3, −*z*+1/3; (x) *x*−*y*+1/3, *x*−1/3, −*z*+2/3; (xi) −*y*+1, *x*−*y*, *z*.
